# A Linearly Deformable Pneumatic Scalable Microgripper for Universal Mid‐Air Micromanipulation

**DOI:** 10.1002/advs.202513679

**Published:** 2025-12-17

**Authors:** Jiawei Yi, Wissem Haouas, Gwenn Ulliac, Kanty Rabenorosoa

**Affiliations:** ^1^ FEMTO‐ST institute Université Marie et Louis Pasteur CNRS BESANCON 25000 France

**Keywords:** 2PP 3D printing, adhesion, micromanipulation, soft robot

## Abstract

The manipulation of microscale components with complex shape like semiconductors, 3D printed microparts, and optical lenses, remains challenging due to strong surface forces and limitations of existing methods. A 3D‐printed soft pneumatic microgripper capable of rectilinear deformation is presented in this paper. It addresses these challenges through a concave design with two operational modes (snap and continuous) and an integrated adhesion‐reducing mask. Fabricated with IP‐PDMS two‐photon polymerisation 3D printing, the microgripper achieves a 40 μm minimum operation diameter and demonstrates a substrate‐free release force as low as 11.1 nN with an adhesion switching ratio of 373 in the normal direction. Combined with rigid alignment, the device enables universal pick‐and‐place over a range of micro‐objects and mid‐air transition of ultralight components (≈1.14 μg) in confined spaces. With over 30000 actuation cycles without performance degradation, this scalable design can execute complex manipulation tasks through a multi‐gripper system as demonstrated.

## Graphical abstract

This work presents a fully 3D printed soft microgripper capable of active adhesion control through a pneumatic system. Its modular design can reach a high adhesion switching ratio of 373 and free release of micro‐objects at 100 μm dimension. The compact, scalable structure enables mid‐air manipulation and release of objects through a multi‐gripper configuration.

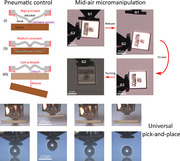

## 1 Introduction

In modern industry, components such as semiconductors, microlenses, and biomedical tissues are becoming increasingly smaller to enable higher integration and functionality in compact environments. However, manipulating and assembling these microscale parts poses a major challenge, as their decreasing weight and the growing influence of small‐scale forces (e.g., van der Waals, electrostatic) lead to increasing instability with reduced size.^[^ ^1^ ^]^

A conventional approach for microscale manipulation is the micro‐tweezer.^[^ ^2^, ^3^, ^4^, ^5^ ^]^ By gripping objects from opposing jaws, this method enables precise handling using a simple end‐effector, allowing manipulation at microscale. However, these mechanisms require a large accessible space and suffer from limited reliability during releasing,^[^ ^1^ ^]^ particularly in object release. Dynamic release strategies such as vibration,^[^ ^6^, ^7^, ^8^, ^9^ ^]^ momentum transfer,^[^ ^10^, ^11^ ^]^ or complex manipulation^[^ ^12^ ^]^ have been explored to mitigate this issue. Alternatively, fluidic actuation,^[^ ^13^, ^14^, ^15^, ^16^ ^]^ often integrated with compliant structures, has been used to develop compact grippers suitable for confined spaces. Nevertheless, these designs generally lack the versatility to reliably pick, orient, and release objects of varying geometries and weights.

**Figure 1:**
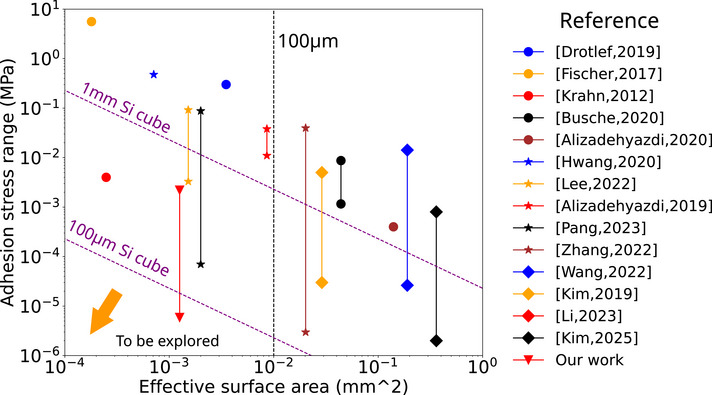
State of the art performance for adhesion control method. The purple line indicates the gravity of a Si cube corresponding to the described dimension. The adhesion stress is the quotient of adhesive force divided by the effective surface area in contact.

Another more universal solution lies in adhesion control. For instance, macroscale adhesion control has been demonstrated using fibrillar structures,^[^ ^17^, ^18^, ^19^, ^20^, ^21^, ^22^ ^]^ where mechanical loading induces shear^[^ ^23^, ^24^, ^25^ ^]^ or vertical^[^ ^26^ ^]^ deformation to modulate contact area. Alternative actuation methods such as pneumatic,^[^ ^27^, ^28^ ^]^ thermal,^[^ ^29^ ^]^ and magnetic^[^ ^30^, ^31^ ^]^ systems have also been explored to enable active control. While effective at macroscopic scales, the collective behavior of these structures cannot be directly translated to the microscale without compromising efficiency. Recent efforts have focused on adhesion control using single^[^ ^32^ ^]^ or limited fiber arrays,^[^ ^33^ ^]^ yet the inherent softness of these structures restricts the achievable adhesion force range, particularly in attaining sufficiently low minimal adhesion forces. A snap‐through mechanism^[^ ^34^ ^]^ has addressed this problem, enabling switching in both contact material and surface area. This approach significantly enhances the adhesion force switching ratio while effectively minimizing the baseline adhesion force. Nevertheless, the device dimensions remain constrained to millimeter‐scale regimes, and its actuation relies on substrate loading and temporary deformation, limiting flexibility and scalability for broader applications. Electrostatic forces^[^ ^35^, ^36^, ^37^, ^38^, ^39^, ^40^ ^]^ have also been widely employed for adhesion control, not without success at microscales. The non‐contact^[^ ^41^, ^42^ ^]^ or rigid contact^[^ ^43^ ^]^ nature of the method yields nice release performance. However, the persistent magnitude of electrostatic interactions at reduced scales compromises manipulation reliability for individual objects. Critically, most existing adhesion control methods constrained by high minimal adhesion forces and actuation specific requirements (e.g., mechanical preload or high voltage differences) depend on substrate assistance, restricting their utility to basic pick‐and‐place operations. The state‐of‐the‐art performances are shown in **Figure** **1**, showing the scaling limitation of the current technology.

Several alternative designs have been proposed to address one or more aspects of the challenge in the past decade. For instance, microscale vacuum grippers^[^ ^44^ ^]^ can achieve a wide range of adhesion forces; however, airflow‐induced instability limits their performance. Capillary grippers^[^ ^45^, ^46^, ^47^, ^48^ ^]^ offer high adhesion forces and self‐alignment capabilities, yet they also suffer from unreliable, slow release and persistent liquid residue issue.

In this work, we present a scalable, fully 3D‐printed soft pneumatic microgripper designed to overcome the aforementioned challenges. The concave structure can be optimised for two distinct operating modes, the snap mode and the continuous mode, suitable for rapid or delicate operations, respectively. Leveraging a simple yet effective design, we achieve a effective operational diameter of 40 μm with fabrication on commercially available printer. The soft polymer material (IP‐Polydimethylsiloxane, or IP‐PDMS) ensures universal and reliable gripping across diverse object shapes and materials. Additionally, we introduce an integrated 3D‐printed mask with minimized contact surface area. The combined system achieves an unprecedented substrate‐free release force of 11.1 nN and a substrate‐release weight of 2.77 nN in the experiment. The adhesion switching ratio in the normal direction is estimated at 373. The device underwent more than 30000 actuation cycles without observable performance loss or structural deterioration. A demonstration of universal pick‐and‐place of micro‐objects in various shapes and materials is performed with a single microgripper. Furthermore, a complex turning manipulation is realised on a microcube with a multi‐gripper system.

## 2 Result

### 2.1 Working Principle of the Soft Actuator

**Figure 2:**
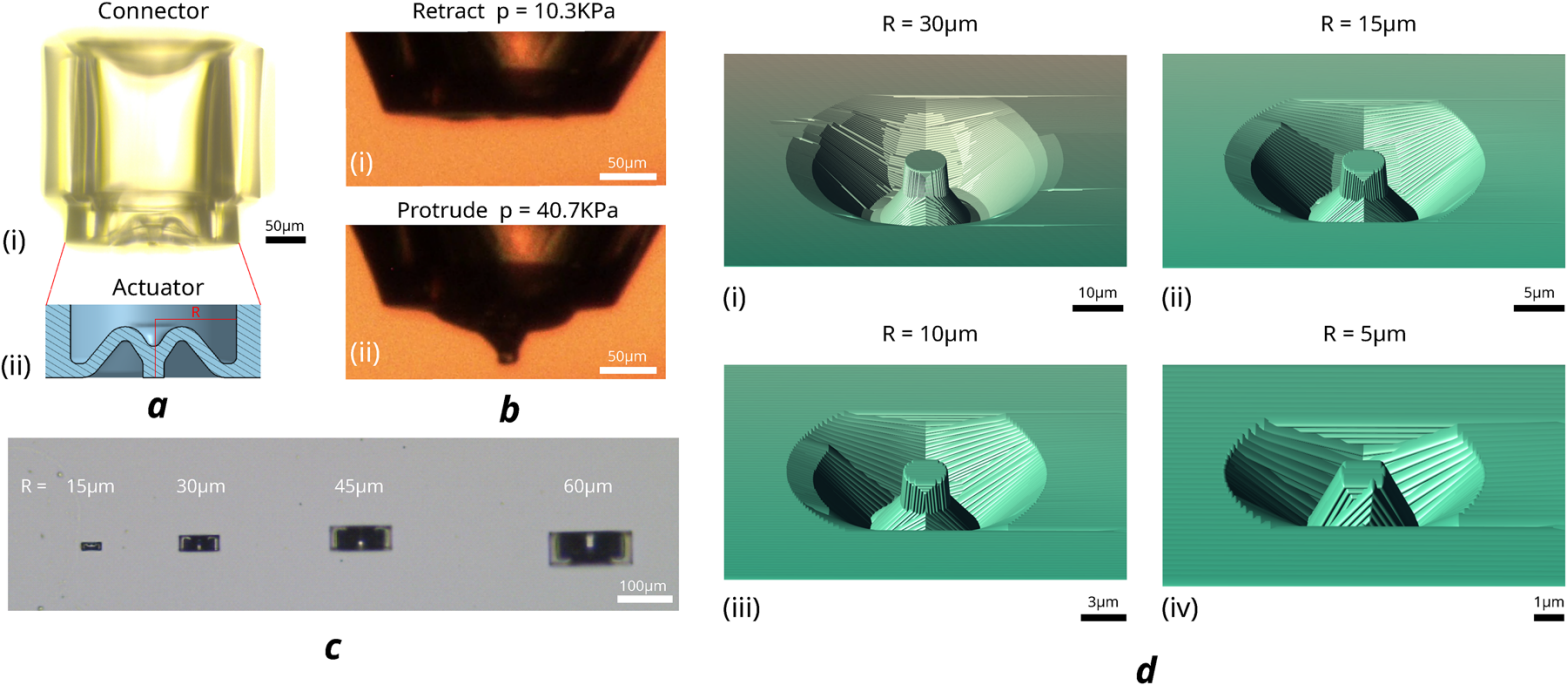
3‐D printed structure of the SSA. ai) Full structure of the actuation system, including the SSA, and the capillary connector, printed with IP‐PDMS. ii) CAD cross‐section of the actuator. bi) Actuator retraction at low pressure of 10.3 kPa. ii) Actuator protrusion at high pressure of 40.7 kPa. c) Scaling fabrication of the actuator. R refers to the radius of the actuator's inner wall. d) Fabrication simulation of further downscaling of the SSA: i) SSA *R* = 30 μm; ii) (i) SSA *R* = 15 μm; iii) SSA *R* = 10 μm; iv) SSA *R* = 5 μm.

This design is inspired by the traditional buckling structures of a soft shell,^[^ ^49^ ^]^ which can achieve a substantial stroke range within a compact dimension. However, inherent elastic buckling is accompanied by an unstable snap‐through effect, which may compromise the reliable manipulation of fragile or lightweight objects. Additionally, a round shell would lose nearly half of its stroke if used as an actuator. To address this, we propose a metastructure, as shown in **Figure** **2**a, in which the top of the shell is curled inward with an extended tip. This soft spike actuator, hereafter referred to as the SSA, increases the utilisable stroke range while maintaining a compact device size. A capillary tube is connected to the end of the SSA, allowing it to be driven by pneumatic pressure. A stable protrude‐retract could be achieved within a relatively small pressure range (under 1 bar) as demonstrated in Figure 2b. When working under the snap regime, the dynamic response time of the actuator's protrusion snap (from 20 to 40 kPa) and retraction snap (from 40 to 20 kPa) is measured to be approximately 0.25s, enabling fast manipulation. The inflation‐deflation process has been repeated more than 30000 times without failure, demonstrating reliability, and robustness over time.

For a perfect hemispherical shell, its critical pressure for snapping could be predicted with Zoelly's equation^[^ ^50^ ^]^:

(1) $$P_{Cr}=\frac{2E}{\sqrt{3(1-\nu^{2})}}{\left(\frac{t}{R}\right)}^{2}$$

with *E*, ν stand for the Young's modulus and Poisson ratio of the material while *t*, *R* refers to the thickness and radius of the shell. The critical pressure is proportional to ${\left(\frac{t}{R}\right)}^{2}$, making it an ideal model for actuator downscaling. The same principle could be applied to our SSA which maintains similar pressure‐strain response. As long as the thickness‐radius ratio stays constant, the theoretical pressure‐strain response would remain unchanged. Though the actuation stroke would scale with the device size as well, the whole model remains theoretically infinitely scalable. In real fabrication, the SSA could be scaled down to 15 μm radius with no detectable structural variation, as shown in Figure 2c. If we further downscale the SSA, as demonstrated in the simulation in Figure 2d (Describe software, Nanoscribe), since the voxel size is constant (approximately 500 nm in size with our configuration), the resolution of the structure would deteriorate drastically, not only causing low repeatability in its actuation response, but also potential imperfection and leakage, ultimately leading to working failure.

**Figure 3:**
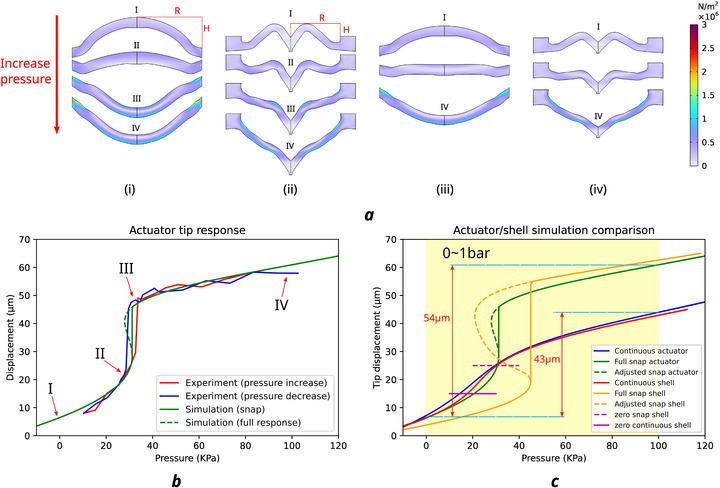
FE simulation of the SSA performance. ai) Shell actuator with *H* = 30 μm, *R* = 60 μm. ii) SSA with *H* = 30 μm, *R* = 60 μm. iii) Shell actuator with *H* = 20 μm, *R* = 60 μm. iv) SSA with *H* = 20 μm, *R* = 60 μm. State I: Pressure=0 kPa, II: before snap, III: after snap, IV: pressure=100 kPa (1 bar). b) Experimental and simulation result of the tip response for a SSA with *H* = 30 μm, *R* = 60 μm. c) Comparison of the simulation results of two working modes.

A FE simulation is conducted to study the detailed deformation response of the shell and the SSA. As shown in **Figure** **3**a, for similar rise *H* = 30 μm and radius *R* = 60 μm, two actuators demonstrated similar working features: two continuous stroke ranges(I–II, III–IV) with a snap (II–III) in between. The actuator's snap effect is validated in experiment with negligible hysteresis between pressure increase and decrease, as shown in Figure 3b. However, if we decrease the rise height to 20 μm, the snap effect fades away and leaves the actuators deforming continuously in the given pressure range (here we choose 0–1 bar), as shown in Figure 3c. Thus, we demonstrated two variations of the SSA, corresponding to two distinct working modes: a snap mode with a long stroke range, suitable for long‐reaching, fast operations at bigger scale, and a continuous mode for smooth, delicate manipulation at smaller scale. Stroke ranges of 54  and 43 μm were achieved in two modes respectively with the given parameters. Further extension of stroke is proven feasible with parameter optimization (see Note S1, Supporting Information).

### 2.2 Release Structure

**Figure 4:**
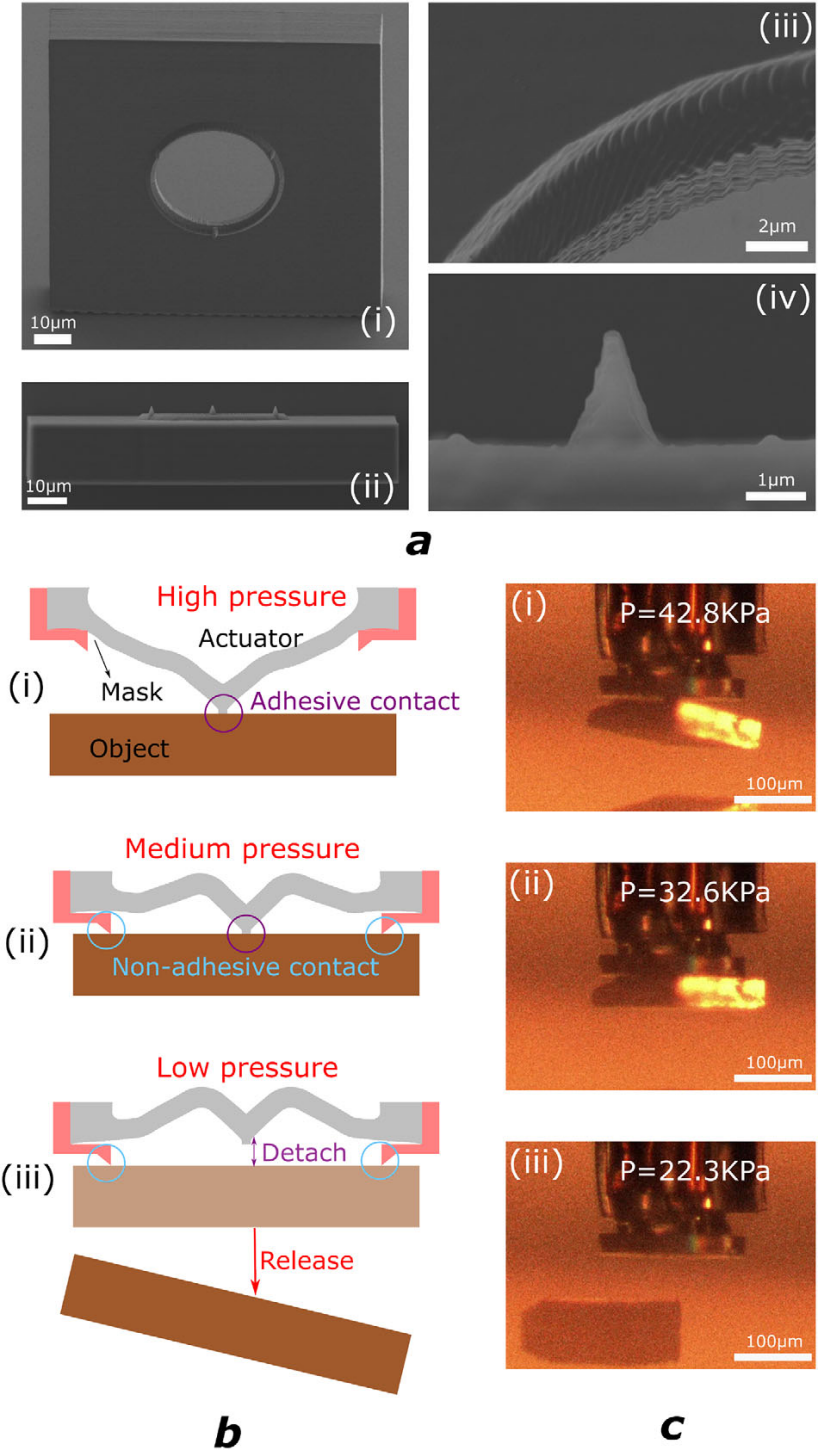
Release structure and mechanism diagram. a) SEM image of the release mask: i) Full structure. ii) Side view. iii) Sharp ring for universal release. iv) Release the thorn. b) Release mechanism: i) High‐pressure attachment; ii) Medium‐pressure object‐orientation. iii) Low‐pressure object release. c) Release process of a Si microchip: i) High‐pressure attachment. ii) Medium pressure object‐orientation. iii) Low‐pressure free release.

While the SSA's adhesive and soft nature is fitting for object pick up, it is not ideal for object release at low force. A release structure is then designed to accompany the SSA to achieve switchable adhesion as well as rigid orientation. The structure is a 3D‐printed mask (IP‐Dip2, Nanoscribe) with highly rigid metal deposited on the surface (here we use Chromium), as shown in **Figure** **4**a. A hole is reserved in the middle for actuator movement, while three thorns are build outside the hole for object orientation and surface release with minimum contact. A sharp ring is reserved for the universal release of irregular surfaces. A minimum ring diameter of 40 μm is paired with our smallest designed SSA (*R* = 15 μm) successfully without hindering actuator motion. When working, the SSA would be inserted into the mask, as shown in Figure 4b. When working under high pressure, the SSA would protrude fully. The soft, adhesive tip would conform to the object surface and bind the object for pick up. When picked, the actuation pressure could be decreased to a medium value so that the non‐adhesive contact of the mask would rigidly orient the object surface while the adhesive tip remains attached. Such medium pressure could be found in the continuous mode as well as the continuous pressure range of the snap mode, as shown in Figure 4c. This orientation mode could effectively overcome the compliant movement induced by the flexibility of the actuator's material and provide alignment precision in the meantime. Similar concept could be found in the capillary gripper design^[^ ^45^ ^]^ as well. Further decreasing the pressure would induce a detachment from the adhesive tip. Then object would be released either with its own gravity or with the adhesion of the supporting substrate. A simple estimation of the release force for each thorn could be given by the DMT model^[^ ^51^ ^]^:

(2) $$F_{MinCr}=2\pi \Delta \gamma_{Cr/Cr}R_{co}$$

where Δγ_*Cr*/*Cr*_ is the work of adhesion and *R*_*co*_ the equivalent radius of curvature for the contact. As can be derived, the release force would depend on the material of the contact surface and the curvature of the thorn tip.

In a real environment, the surface roughness of the tip‐object contact would also influence the release force, which renders the quality of fabrication and object surface the two main factors influencing the release force. As can be observed in Figure 4a, the resolution of the fabrication is under 1 μm and the formed surface on the release tip is optically smooth under SEM. As for the object surface, for a rigid–rigid contact between the metal thorns and the released object, the increased surface roughness would result in a decrease in the Van der Waals force since the mean contact distance increases with the roughness.^[^ ^52^ ^]^ So in later characterizations, we use smooth object surfaces to demonstrate the upper limit of the release force. In our experiment, a substrate‐free release of 11.1 nN was achieved, while a substrate‐facilitated release of 2.7 nN was achieved on a Cr‐coated substrate, both with Cr deposited polymer cubes (metal‐metal contact). This shows an extremely low release force for our design, enabling further flexible operations for micro‐objects.

### 2.3 Microgripper Characterization

**Figure 5:**
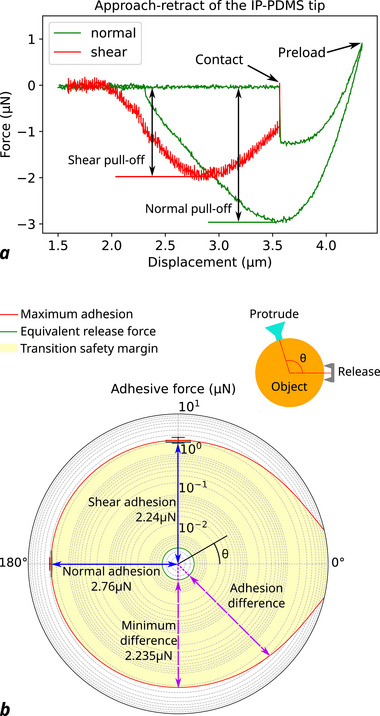
Adhesive force characterization. a) Approach‐retract process of the normal/shear adhesion of the SSA tip. b) Analysis diagram for mid‐air transition of an object.

The maximum adhesive force of the SSA's soft tip (actuator *R* = 15 μm, tip diameter 7.5 μm) is characterized with the approach‐retract process, as shown in **Figure** **5**a. Both shear and normal directions are measured with ten repetitions using a FEMTO tools sensor with *Si* tip. In shear direction, the pull‐off force is measured right after contact jump‐in, while the normal direction is measured after a stable, relatively small preload (1μN) is applied. Both directions could achieve pull‐off forces above 2μN, which is significant compared to the release force measured before. The adhesion switching ratio in the normal direction is estimated to be 373 (see Note S2, Supporting Information). This large and repeatable adhesion transition demonstrates the potential for mid‐air object transfer. As analyzed in Figure 5b, when releasing an object with another microgripper's tip attached in a θ angle, the release force is always notably lower than maximum adhesion force generated for detachment. The switch ratio between the maximum adhesion and release force is always higher than 200 for any θ direction, ensuring an omnidirectionally stable and reliable transition.^[^ ^53^ ^]^

### 2.4 Manipulation Performance

**Figure 6:**
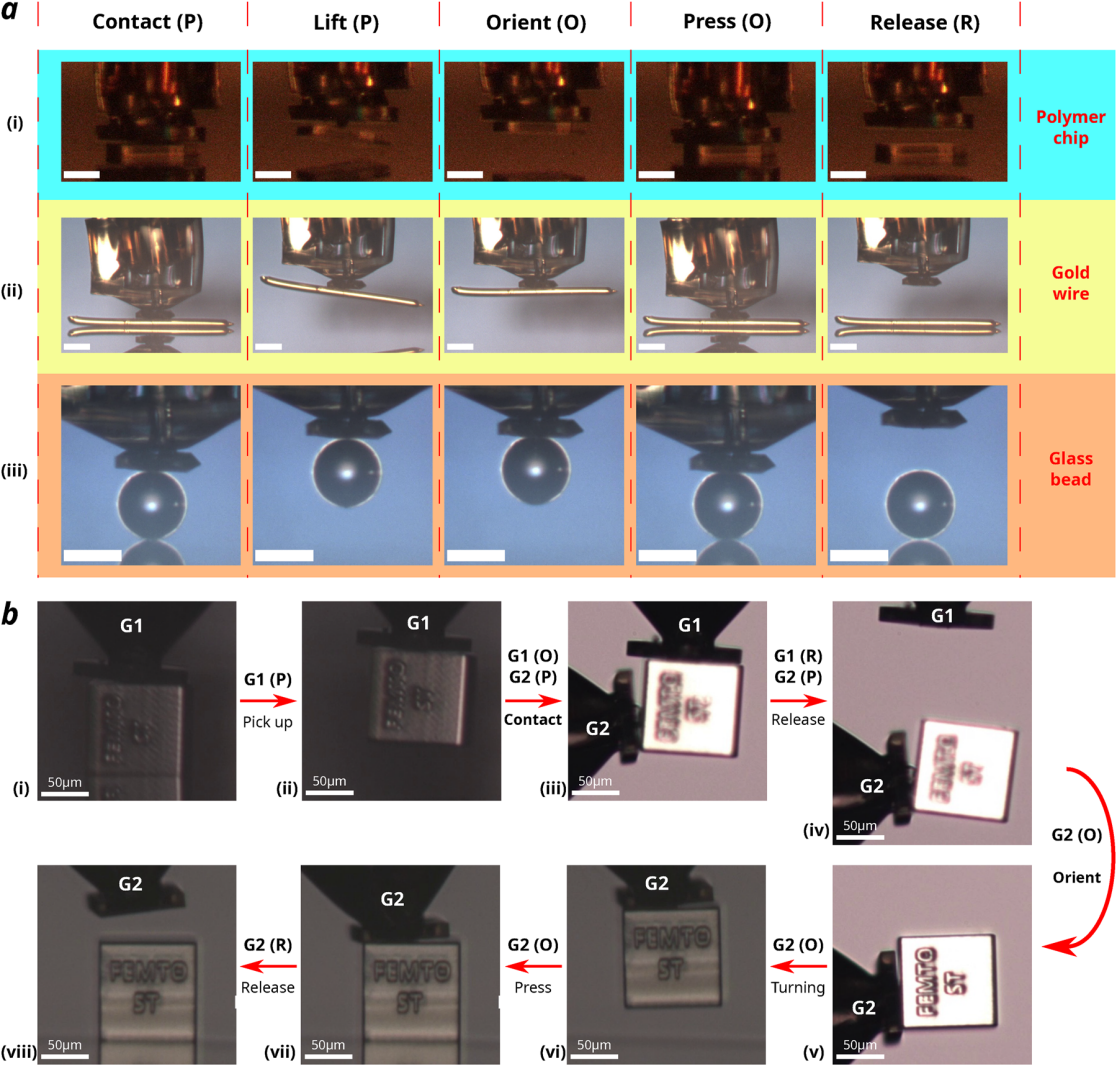
Object manipulation performance. a) Pick and place of micro‐objects on a glass substrate: i) Polymer (IP‐S) chip of 200 × 200 × 25 μm (SSA *R* = 60 μm). ii) Metal (gold) wire of 500 μm length and 25 μm diameter (SSA *R* = 15 μm). iii) Glass bead of 100 μm diameter (SSA *R* = 15 μm). Scale bars refer to 100 μm. b) Mid‐air manipulation of a 100 × 100 × 100 μm IP‐S block with Cr deposition on a Cr deposited substrate. G1, G2 refer to two identical microgrippers. i) First actuator‐object contact. ii) Object pick up. iii) Second contact. iv) Release from first contact. v) Orientation. vi) 90° rotation of the object. vii) Substrate‐object contact. viii) Object release. Microgripper's working state is presented as **P** (protrude), **O** (orientation) and **R** (release). SSA radius *R* = 15 μm.

Since the adhesion mechanism of the microgripper relies on mechanical contact, its manipulation performance depends entirely on the quality of the contact. It enables the microgripper to reliably pick and release objects of diverse materials, including metals, glass, and polymers, as shown in **Figure** **6**a. The release of these objects is all realised on a non‐adhesive glass substrate. The actuator's compliance facilitates robust initial adhesive contact across various surface geometries, such as flat, spherical, and cylindrical shapes, while its orientation mode ensures rigid and precise object alignment. These characteristics make the microgripper a versatile and practical solution for universal applications.

Beyond conventional pick‐and‐place operations, the SSA's omnidirectional high adhesion and the low release forces of the mask enable in‐air object transitions, facilitating mid‐air multi‐gripper manipulation. This capability expands the range of possible micro‐object manipulations—including flipping, rotating, and precise alignment—as demonstrated by the 90° rotation of a Cr‐coated polymer cube in Figure 6b.

## 3 Discussion

Here, we demonstrate a 3D‐printed soft microgripper designed for micro‐object manipulation. As shown in Figure 6b, the high adhesion switching ratio enabled reliable pick‐and‐place operations for a wide range of micro‐objects with various geometries and materials. Its omnidirectional adhesion and low preload requirement allow for stable mid‐air transitions, enabling flexible in‐air manipulation. Notably, the microgripper's soft tip and high compliance eliminate the ‘jumping’ effect commonly observed with rigid multi‐contact microgrippers, ensuring stable object handling. Furthermore, since each microgripper requires only mechanical contact with one surface, the manipulation process requires minimal workspace compared to traditional MEMS tweezers, significantly reducing operational complexity, enabling it to reach confined spaces. As there is negligible physical interference between individual mechanical contact compared to electrostatic (field disturbance) or capillary (liquid fusion) contacts, a multi‐gripper system is proven viable for performing complex manipulation of micro‐objects.

**Figure 7:**
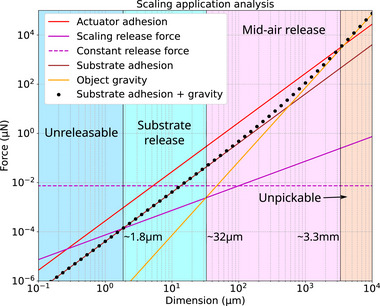
Scaling analysis. The adhesive force of SSA, release mask, substrate on a designated object, and their scaling effects. The reference dimension for the smallest microgripper (*R* = 15 μm) is set to be 100 μm. The object is defined as a Chromium cube with dimension as its length. The referred substrate is defined as a smooth Cr deposited non‐adhesive surface.

On the scalability of the pick and release mechanism, we have characterized the maximum adhesion with approach‐retract process and minimum release force with the smallest mid‐air release weight. To set the reference dimension of the characterized microgripper (*R* = 15 μm) as 100 μm, an adhesion force map concerning the adhesion of the SSA, the release mask and substrate contact could be constructed to analyse the applicable dimension for our current design, as shown in Figure 7 (modelling detailed in Note S3, Supporting Information). As the tip and the substrate scale with their contact area, the tip adhesion would be constantly higher than the substrate adhesion. The factor determining whether the object could be successfully picked up lies solely in the weight of the object, which is usually insignificant at microscale. Even for high‐density material such as Chromium, its weight would only break the tip's adhesive contact at millimeter scale (estimated at 3.3mm in the analysis). As long as the object weight is higher than the mask's release force, the object could be released in mid‐air without the facilitation of the substrate. The dimension limit of such mid‐air release zone is estimated to be around 32 μm under the assumption that the release mask's fabrication resolution would scale down with the dimension. If we keep the fabrication resolution as a constant (3D printing method), the limit would be slightly higher but still under 100 μm. As the object weight decreases, the release would require the assistance of the substrate. The substrate does not need to be adhesive as the release relies mainly on the contact area difference. A lower limit of 1.8 μm in dimension is estimated for substrate release before the release force surpasses the substrate adhesion. It should be noted that the metallic deposition on the release mask would induce an electrode transfer once in contact with the object. If the material is conductive, as assumed in the analysis, the residual like charge would generate electrostatic repulsion between the mask and the object, making the object easier to be released in air. However, for semi or non‐conductive material, a non‐predictable electrostatic force should be considered when analyzing the pick‐and‐place capabilities. But as demonstrated in the previous section, materials like polymer or glass could still be released on non‐adhesive substrate reliably.

With the acquired results, we compare our work with the state‐of‐the‐art adhesion‐based grippers to show the advantages and limits, as shown in **Table** **1**. For pure contact‐based methods, the manipulation dimension is close to our work,^[^ ^32^, ^34^ ^]^ with usually an extremely high switch ratio (generally exceeding 1000). But the fabrication of these structures are usually one‐piece molding or printing, which prevented the further optimization of the adhesive force. This resulted in a much higher adhesion stress at release than our work. For example, the lowest release adhesive stress by pure contact is 2.97 Pa on substrate, which is even higher than our mid‐air release stress. The physical‐based methods could efficiently optimize the contact to achieve a much lower release force, which renders small object size (nanowire with electrostatic gripper^[^ ^43^ ^]^) or low release stress (1.8 Pa in capillary gripper^[^ ^48^ ^]^). But these physical phenomenons have their own limitations. The electroadhesion would generate high residual adhesion, which limited the release performance, making the device more suitable for transfer printing. Capillary force has avoided the residual adhesion problem and has achieved the lowest release adhesion stress in the literature. But the capillary adhesion could not be actively “switched off”, so the current control method is only natural evaporation of the liquid, taking generally more than 10s.

**Table 1:** Comparison of the state of the art, all the parameters are taken from the best performance in the literature in the context of repeatable pick‐and ‐place. The smallest dimension refers to the dimension of the smallest object demonstrated in a pick‐and‐place.

Reference	Principle	Smallest dimesnion [$\mu m^{2}$]	Switch ratio	Minimum adhesion stress (Pa)	Response time (s)	Mid‐air release
^[^ ^32^ ^]^	Contact/suction	1 × 10^4^	×1254	70	/	No
^[^ ^34^ ^]^	Contact	2 × 10^4^	>×10^4^	2.97	/	No
^[^ ^43^ ^]^	Contact/electro	2.89 × 10^4^	×166	30	/	No
^[^ ^48^ ^]^	Contact/capillary	3.6 × 10^5^	×444	1.8	>10	Yes
Our work	Contact	1 × 10^4^	×373	1.11	∼0.25	Yes

In comparison, our work, by the incorporation of the modular design, is able to optimise both the maximum and minimum adhesion of the system in separate processes. This has led to an extremely low release adhesion while retaining a highly adhesive structure for pick‐up. The concave design extended the stroke of the actuator significantly, leaving enough work space for the design of the release mask and the adhesion switch mechanism, rendering the assembly and switching process reliable and effective. The modular design also permits the substitution of the release mask (as shown in Movie S5, Supporting Information), effectively lowering the influence of environmental contamination. The active control through pneumatic pressure has enabled fast operation while avoiding introducing new source of forces. The snap‐continuous working regime leaves us with more flexibility to adapt to more complex application conditions. However, as a prototype, the further optimization of the structure's adhesive forces, whether it is minimum or maximum, could be explored with existing methods validated in the literature, such as the PDMS‐dipping for a more adhesive tip^[^ ^34^ ^]^ and the carbon nanotube surface for diminished contact area.^[^ ^43^, ^48^ ^]^ This would leave an interesting prospect for future work.

## 4 Experimental Section

### Microgripper Fabrication and Assembly

**Figure 8:**
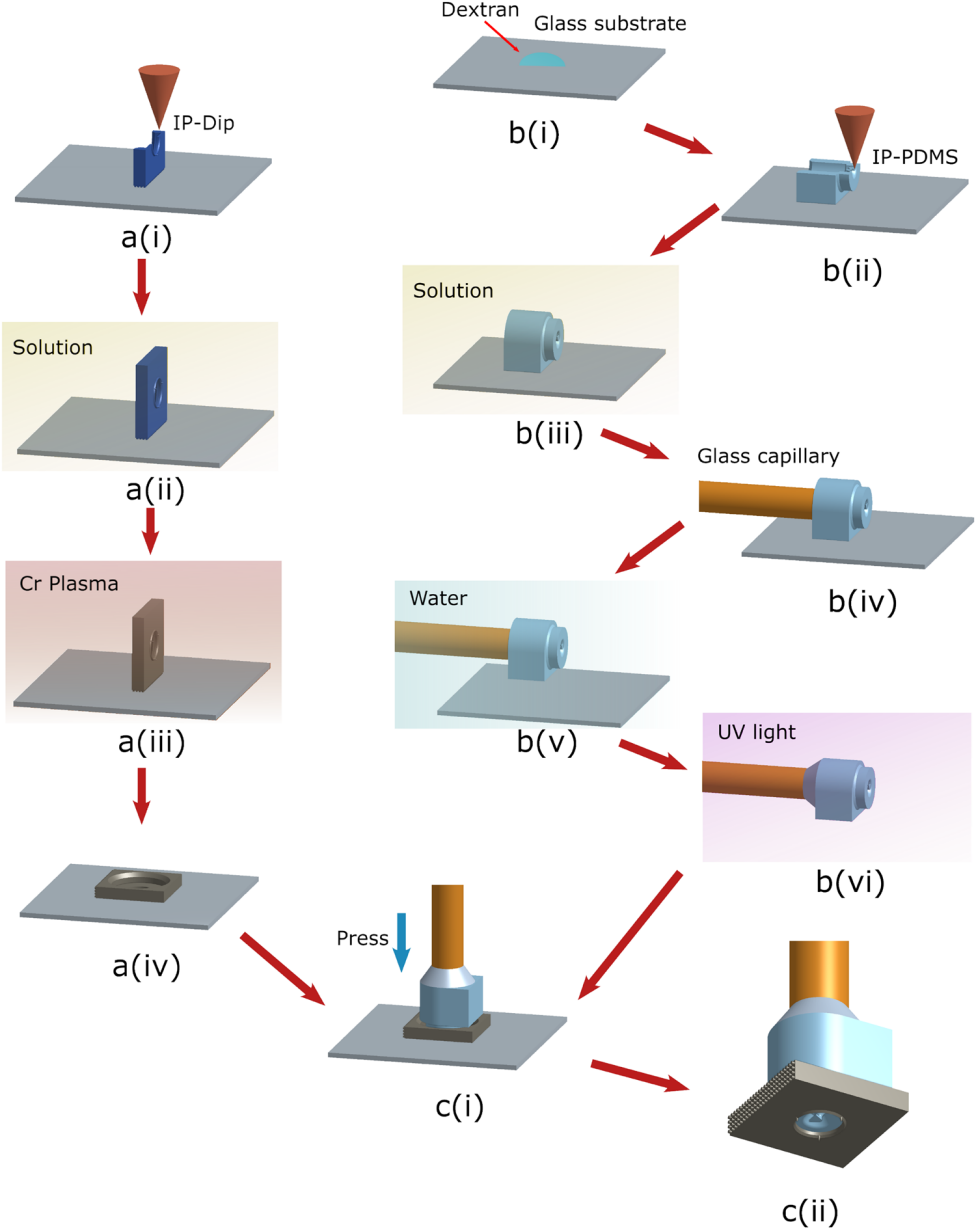
Fabrication and assembly diagram of the microgripper. ai) 2PP printing for mask. aii) Solution wash. aiii) Cr deposition. aiv) Manual tipping. bi) Spin coating of dextran. bii) 2PP printing for SSA. biii) Solution wash. biv) Insert capillary tube. bv) Release in water. bvi) UV glue sealing. ci) Assembly. cii) Final product.

As shown in Figure 8, the fabrication of both SSA and mask were done mainly with 2PP 3D printing (Nanoscribe, Photonic Pro. GT+). For the printing of the SSA body, a dextran layer was first spin‐coated (3000 rpm, 3000 rpm/s, 30 s) onto a glass substrate (ITO‐coated soda lime) for structure release. The body was then printed onto the substrate with IP‐PDMS (IP‐PDMS photoresin, Nanoscribe) and 25x objective (70% power scaling, 90 contour/solid laser power, 50000 μm s^−1^ contour/solid scan speed). After washing with IPA (two 10‐min baths in individual solutions), the connector part was inserted with a glass capillary (rim diameter 160 μm). The structure was then soaked in water and released as dextran dissolves. UV glue (Norland optical adhesive 63, FT Polymer) was applied to seal the connection. It should be noted that the mechanical properties of the IP‐PDMS material could change after a long or strong UV exposure, even though no significant influence was observed on the device after a 5‐day exposure in natural light (see Note S7, Supporting Information). As the pressure‐displacement response of the SSA could be sensitive to such influence, the sealing of the connection should guarantee a short exposure of UV light. If the repeatability of the mechanical properties was of vital importance for a specific application, an alternative approach could be suggested for sealing, where a PDMS (Sylgard 184, Dow Chemical) of 10:1 polymer/curing agent mixture was applied instead and let it cure over time with no heating or UV light applied.

The release mask was printed with IP‐Dip2 and 63x objective directly onto a fused silica substrate. After printing, the mask was then washed (12 mins in PGMEA and 5 mins in IPA) and deposited with an estimated 25 nm thickness of Chromium (Leica, EM ACE 600). Afterwards, the mask was manually released and the SSA was pressed into the mask (as shown in Movie S5, Supporting Information). The two parts were then firmly assembled with adhesion.

### Force Measurement

The measurement of adhesive force was conducted with FT‐MTA03 platform from FemtoTools AG with a microforce sensing probe (FT‐S20000 NI, Si flat punch, 50 nN resolution). For normal approach‐retract, the compression test mode was activated, where the sensor tip end would press a soft tip same size as the SSA, placed vertically. After reaching a preload, the sensor tip would retract at a constant speed until detachment. The preload here was set to be 1 μN and retract speed 0.1 $\mu ms^{-1}$. For shear measurement, the sensor tip's vertical side (flat) was moved slowly toward the soft tip, which was placed horizontally, until the sensor surface jump into contact (movement resolution was set at 0.1 μm). Then, the tip was retracted vertically at 0.1 $\mu ms^{-1}$ until the force measurement stabilizes. It was then retracted horizontally to ensure complete detachment. All subsequent repetitions begin from the same initial position (detailed in Figure S4, Supporting Information).

### Displacement Tracking

The displacement of the SSA tip was measured using the OpenCV library. A video capturing the pressure‐displacement response of the SSA was recorded, and individual frames exhibiting stable deformation were extracted and analyzed using a custom OpenCV‐based algorithm. The protruding displacement was defined as the linear distance between the tip and the SSA's outer boundary. To automate the detection of these points, a customized threshold and aperture size were applied, enabling edge detection to identify the two boundary endpoints and the spike tip. The distance was initially computed in pixels and subsequently converted to physical units using a calibrated reference length (detailed inFigure S5, Supporting Information).

## Conflict of Interest

The authors declare no conflict of interest.

## Supporting information



Supporting Information

Supporting Information

Supporting Information

Supporting Information

Supporting Information

Supporting Information

## Data Availability

The data that support the findings of this study are available from the corresponding author upon reasonable request.
