# Supplementary material for: A Linearly Deformable Pneumatic Scalable Microgripper for Universal Mid‐Air Micromanipulation
Source: Adv Sci (Weinh). 2025 Dec 17;13(10):e13679. doi: 10.1002/advs.202513679 (PMC12915121; doi:10.1002/advs.202513679)
Supplement: Supplementary file 1 — Supporting Information [file ADVS-13-e13679-s003.pdf]

ADVANCED SCIENCE Open Access

Supporting Information for

A Linearly Deformable Pneumatic Scalable Microgripper for Universal Mid-air Micromanipulation

Jiawei YI, Wissem HAOUAS, Gwenn ULLIAC, Kanty RABENOROSOA

Wissem HAOUAS.
wissem.haouas@femto-st.fr

This PDF file includes:

Supporting text
Figs. S1 to S8
Legends for Movies S1 to S5
SI References

Other supporting materials for this manuscript include the following:

Movies S1 to S5

Supporting Information Text

Supplementary Note 1. Structure parameter analysis

The design of the SSA lies on three key parameters: the height h, the radius r of the lift and the thickness of the membrane Th. In shallow shell theory (1, 2), the snapping-continuous regime of a shell (or in our case, the concave structure) is determined by the energy barrier between two stable states (protrude and retract). This energy barrier is primarily determined by the thickness of the shell. The influence of the thickness could be quantifies with Zoelly's theory (3) or with the dimensionless Föppl–von Kármán number (4):

$$\gamma = 12(1-\nu^2)(\frac{R_0}{Th})^2 \quad [1]$$

in which R_0 is the rest radius and ν the Poisson ratio. The energy barrier would increase with the Föppl–von Kármán number, meaning that a thinner membrane is more prone to snapping while a thick one is more prone to continuous movement.

Concerning the lift height, the energy barrier would generally increase as the ratio $\frac{h}{R_0}$ increases, which could also be induced from the shallow shell theory (1). In our case, if we fix the radius of the structure R, as we increase h, the analogical rest radius R_0 would decrease, leading to a higher energy barrier. The lift radius r is a parameter specific to our design so we will validate its influence with the results in simulation.

Here we further validate the influence of these three design parameters with FEM simulations. As described in the main text, the deformation-pressure should remain unchanged as long as the model is fully scaled in all 3 dimensions. So here we base the parameter variation on a base design of R=60 µm, r=20 µm, h=30 µm and Th=10 µm, as shown in Fig.S1**a**.

Lift height. The height of the lift, in the context of a shell actuator, indicates the shallowness of the shell. As shown in Fig.S1**b**, with the lift height increasing from 15µm to 35µm, we can see that the structure would evolve from a continuous motion regime into a snap regime, with the maximum stroke range increasing with the height. The snap-initiation pressure for snap, once in the snap regime, would increase with the height as well. It could also be observed that the maximum stroke at 1 bar is almost proportional to the height, making it a design indicator for achievable stroke range.

Lift radius. The radius of the lift would influence the concentration of stress in the structure as well as its displacement response. As shown in Fig.S1**c**, as we increase the radius from 15 µm to 30 µm, the snap-initiation pressure and the maximum stroke range would increase slightly. However, as its influence is insignificant compared to other parameters, it is suggested to choose a medium value (in our case 20 µm) to avoid stress concentration on the edge of the membrane.

Membrane thickness. The thickness of the membrane is one of the most important parameters to consider as it is not only influenced by design but also heavily by fabrication process. As shown in Fig.S1**d**, a decrease in thickness would shown a drop in required actuation pressure for any given displacement. Even though the motion evolves from a continuous to a snap-through regime, the required initiation pressure also decreases, accompanied by a significant increase in maximum stroke. This makes thinner membranes more favourable in terms of energy efficiency and ease of control. However, during fabrication process, as shown in Fig.S2, a lean membrane could cause considerable deformation (Th=5 µm) or even complete failure (Th=2.5 µm) as its structure is not resilient enough to withstand the thermal shrinkage of the polymer (5). So it would be recommended to design the structure with sufficient thickness for fabrication precision.

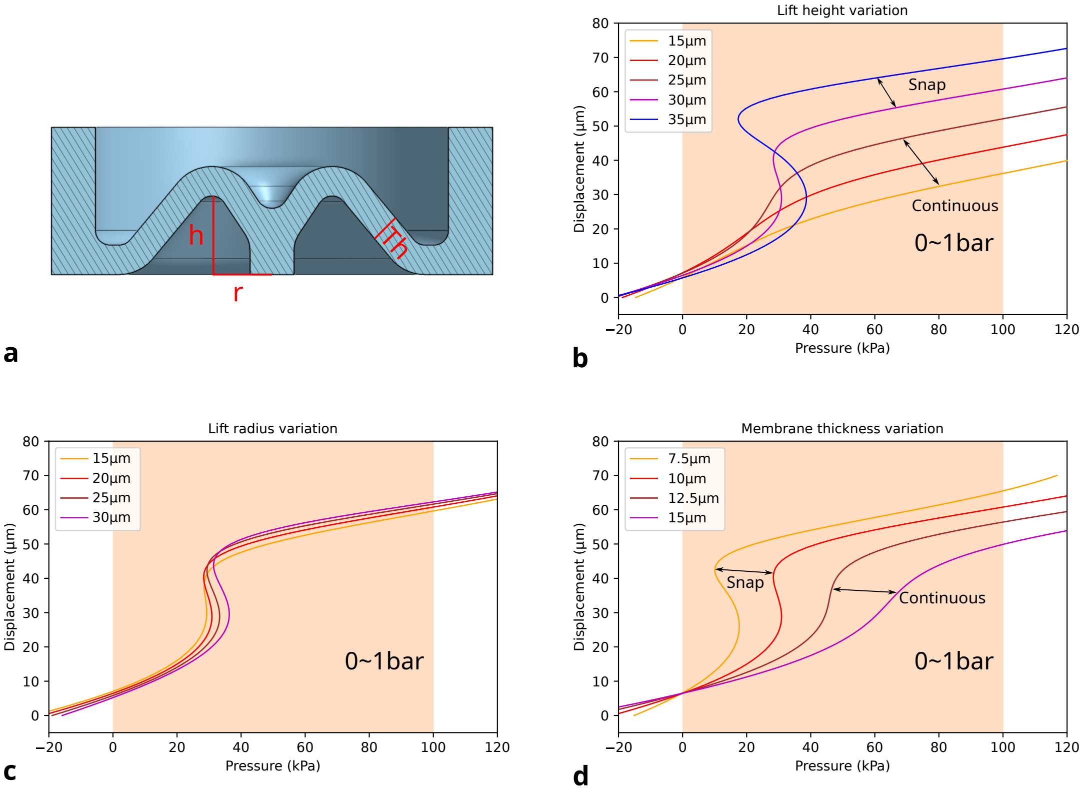

Fig. S1. FEM simulation for pressure-displacement response based on parameter variation. a CAD crossssection of the SSA with the parameters in question. **b** P-D response of lift height variation. **c** P-D response of lift radius variation. **d** P-D response of membrane thickness variation.

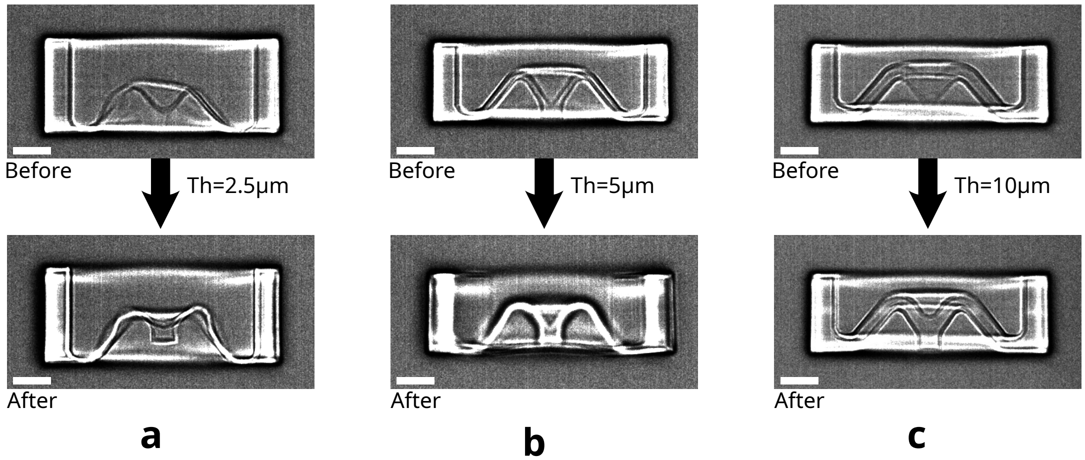

Fig. S2. Live images of fabrication failure due to thermal shrinkage in Nanoscribe. **a** Thin membrane (Th=2.5 μm) failure. **b** Medium membrane (Th=5 μm) deformation. **c** Thick membrane (Th=10 μm) fabrication with no obvious deformation. Scale bars refer to 25 μm

Supplementary Note 2. Switch ratio calculation

As the adhesion maximum and minimum of the system are conducted on two different contacts (Si-polymer contact and Cr-Cr contact), a normalisation of forces is required to calculate the adhesion switch ratio. As we take the maximum adhesive force as the base of our comparison in Fig.**5b**, here we only calculate the equivalent minimum adhesion of the release mask on a Si surface. For the equivalent Cr-Si contact, we can estimate the pull-off force with the DMT model(6):

$$F_{MinSi} = 2\pi\Delta\gamma_{Cr/SiO_2}R_{co} \tag{2}$$

As the Si tip in the FEMTO tools is exposed in the air, we use the work of adhesion for a Cr-SiO_2 contact instead for accuracy. Thus we further derive:

$$F_{MinSi} = F_{MinCr}\frac{\Delta\gamma_{Cr/SiO_2}}{\Delta\gamma_{Cr/Cr}} \tag{3}$$

According to Dupré's equation for work of adhesion(7):

$$\Delta\gamma_{Cr/SiO_2} = \gamma_{Cr} + \gamma_{SiO_2} - \gamma_{Cr/SiO_2} \tag{4}$$

So we have a conservative estimation of the equivalent release force:

$$F_{MinSi} < F_{MinCr}\frac{\gamma_{Cr} + \gamma_{SiO_2}}{\Delta\gamma_{Cr/Cr}} \tag{5}$$

Here we substitute the real values: $\gamma_{Cr} = 2.1J/m^2$, $\gamma_{SiO_2} = 0.7J/m^2$, $\Delta\gamma_{Cr/Cr} = 4.2J/m^2$, the equivalent release force is calculated to be no higher than 7.4 nN and the switch ratio larger than 372.97.

Supplementary Note 3: Scaling analysis

The scaling of the maximum/minimum adhesion of the system depends on the contact model we choose. For the normal contact between IP-PDMS tip and Cr object, we have the expression based on work of adhesion:

$$F_{Ad} = A\Delta\gamma_{Cr/IP-PDMS} \tag{6}$$

where A refers to the contact surface area. Such model applies to substrate adhesion as well. So we have the two forces scaling with D^2, D indicating the referred dimension length.

For release force of the mask, we have the DMT model(6) same as presented in the main text:

$$F_{MinCr} = 2\pi\Delta\gamma_{Cr/Cr}R_{co} \tag{7}$$

As we have mentioned, if we keep the fabrication precision constant, the release force should be expected to remain unchanged as the end tip would remain a curved tip with a constant curvature radius. But if we assume the fabrication precision (here equivalent to R_{co}) scales with the dimension, then the release force would scale with D. In the above calculations, a smooth contact surface with negligible roughness is assumed, which is common for microobjects such as Si chips, glass or polymer parts. However, if we are to apply the model on specific object or conditions, additional model modifications might be required to improve accuracy.

Supplementary Note 4: Experiment set up details

The experiment stage consists of 3 parts: motion system, pressure system and feedback system. The motion of the system is controlled with a robotic arm developed in-house with 3 linear actuators (SLL-1C42 and SLC-2490, SmarArc), 2 gniometers (SGO-77.5 and SGO-60.5, SmarArc) and one rotation actuator (SR-5714, SmarArc). The sensor resolution is less than 10 nm and 1 µ°. The adhesion system is inserted with a glass capillary tube and fixed onto the arm with a 3D printed connector. The visual feedback system consists of two cameras with microscopic lenses. The connector is attached with two Silicon mirrors for better light reflection. The capillary tube is connected to a pneumatic control system (OB1 MK3+, Elveflow) coupled with a vacuum pump (VP18 Plus, LabTech) for pressure control.

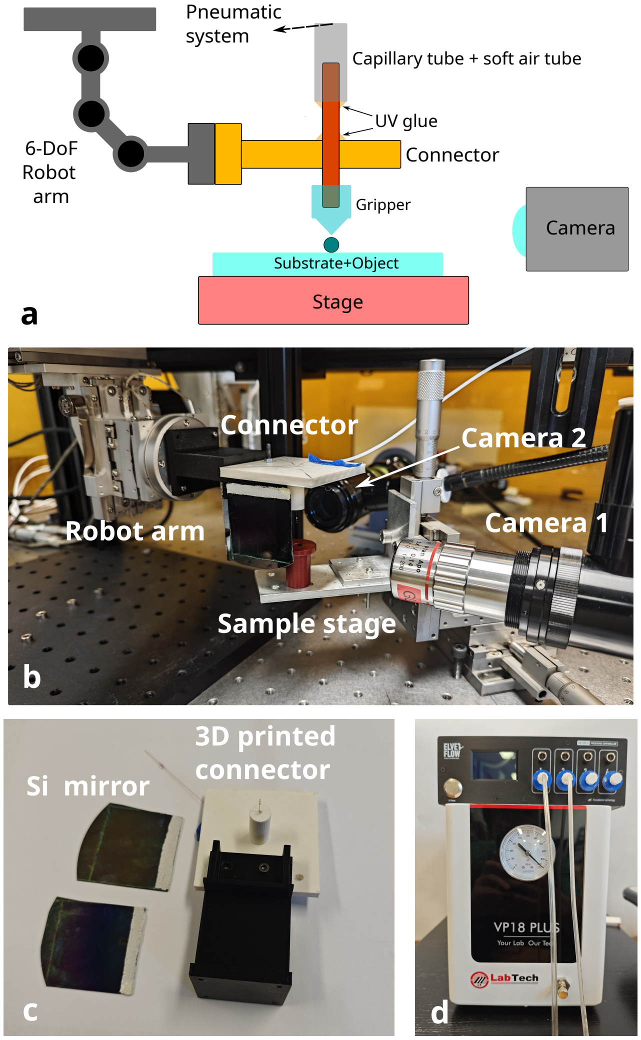

Fig. S3. Experiment set up a Diagram of the experiment set up. **b** General stage set up. **c** Connector structure. **d** Pneumatic controller.

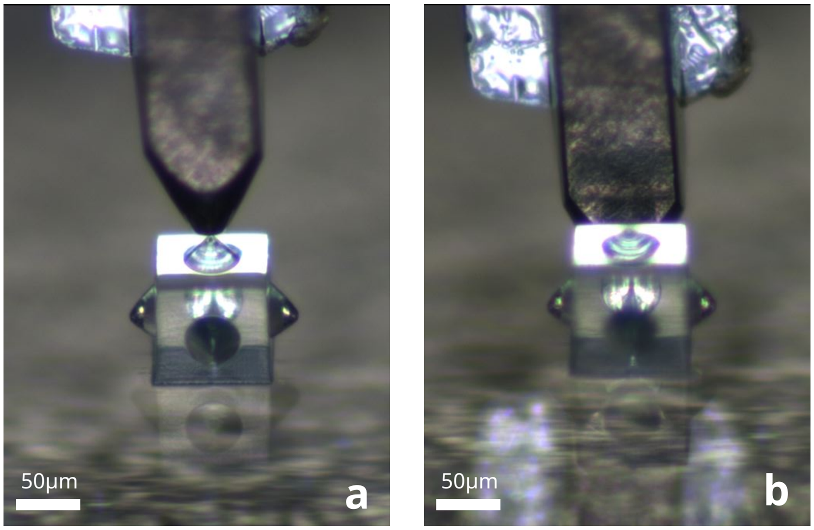

Fig. S4. FEMTO tool set up for adhesion characterization. a Normal contact between the Si tip and the fabricated IP-PDMS tip. **b** Shear contact.

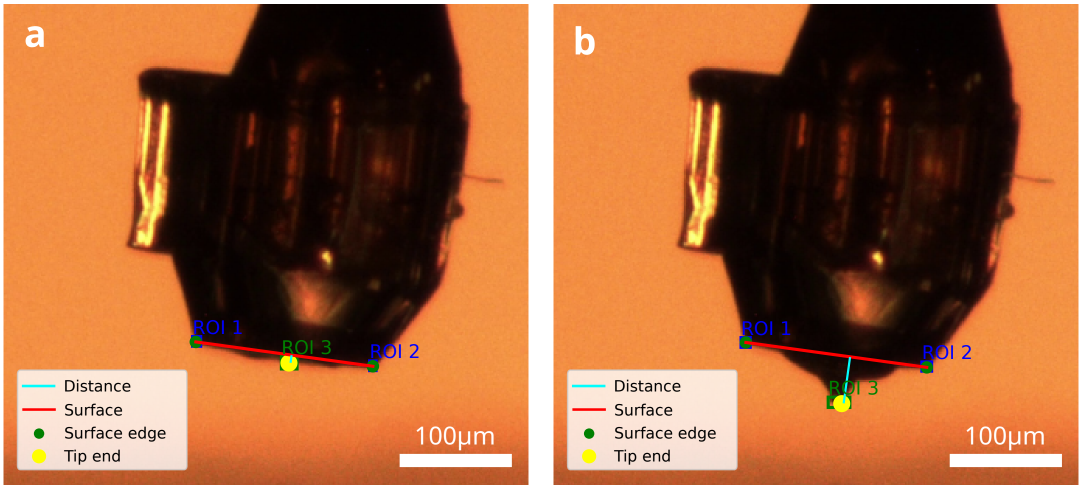

Fig. S5. Processed image for displacement tracking. The ROI refers to the region of interest for edge detection. a Measurement of a retracted tip at P=15.3 kPa. **b** Measurement of a protruded tip at P=61.1 kPa.

Supplementary Note 5: Performance repeatability

The performance repeatability of the microgripper can be divided into three aspects: (i) pressure–displacement deformation consistency across multiple samples, (ii) endurance performance over repeated actuation cycles, and (iii) surface adhesion repeatability.

The snapping pressure of three SSAs samples (R= 60 µm) are measured to be 29.6 kPa,32.6 kPa and 32.5 kPa in retraction with 1kPa stepping. This repeatable threshold is observed to be consistent over 30000 repetitions, demonstrating high repeatability and consistent fabrication quality.

For surface adhesion repeatability, a series of indentation experiments was conducted on an IP-PDMS pad (200×200×30 µm) fabricated under the same conditions as the SSA actuator. The indentation is performed with FEMTO tools (FT S20000, glass hemisphere r=25 µm, 50 nN resolution). Loading and unloading velocity are set at 0.2 and 0.1 µm/s with a 4 s stay after reaching the preload of 100 µN. The indentation is performed on 10 consecutive positions on the pad with 10 µm gap in between, as shown in Fig.S6**a**. The deviation of the pull-off force is 0.61 µN, showing a deviation rate of 3.8%, demonstrating high adhesion repeatability.

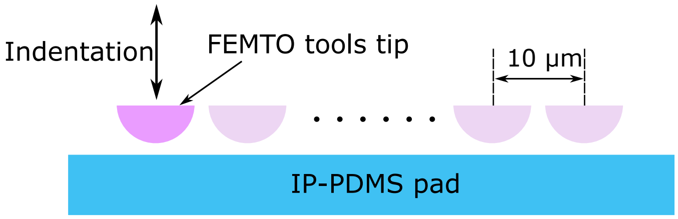

a

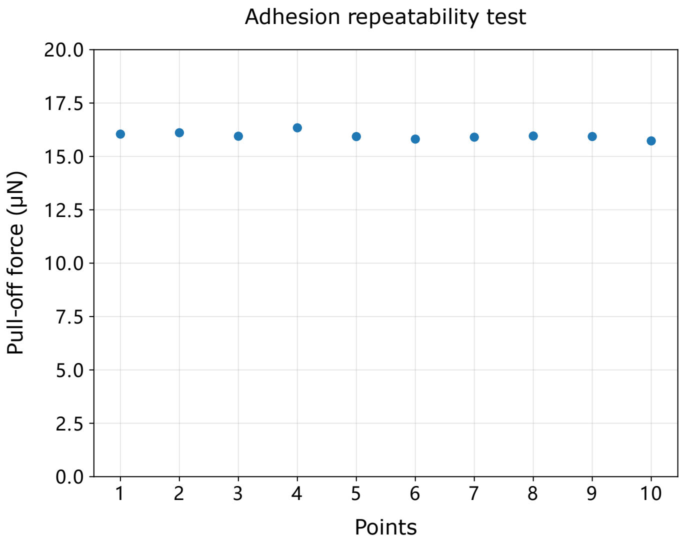

b

Fig. S6. Indentation experiment for adhesion repetability. a Schematics of the indentation experiment on 10 points. **b** Pull-off force result.

Supplementary Note 6: Environmental influence

The manipulation of microobjects is generally conducted in controlled environment (cleanroom or equivalent environment). However, it is also necessary to qualitatively test the possible adhesion degradation on the material surface caused by environmental influence (contamination, natural light, temperature change, etc.). To this end, we have conducted a 5-day long experiment, during which we perform the indentation experiment in **Supplementary Note 5** every day. But instead of 10 positions, we do the indentation at the same position for 10 repetitions. The sample is left in an open, uncontrolled environment (room temperature 20±5°C, humidity 63%) perceptible to natural light and air dust. The test result is shown in Fig.S7. The pull-off force has decreased 1.85 mN over 5 days, approximately 12.8% of the overall force. This shows that the SSA has a reasonable resistance to environmental influence. But if it is to be used for a long period of time, it is recommended to clean the tip surface (solution soaking or laser-cleaning) when there is accumulated contamination.

Another environmental consideration concerns potential adhesive contamination on the release thorns, which may increase the release force and compromise manipulation performance. Due to the small scale of the release mask, direct cleaning is challenging. Nevertheless, the modular design of the gripper enables easy replacement of the mask, as shown in movie **S5**, ensuring that the release performance can be maintained as needed.

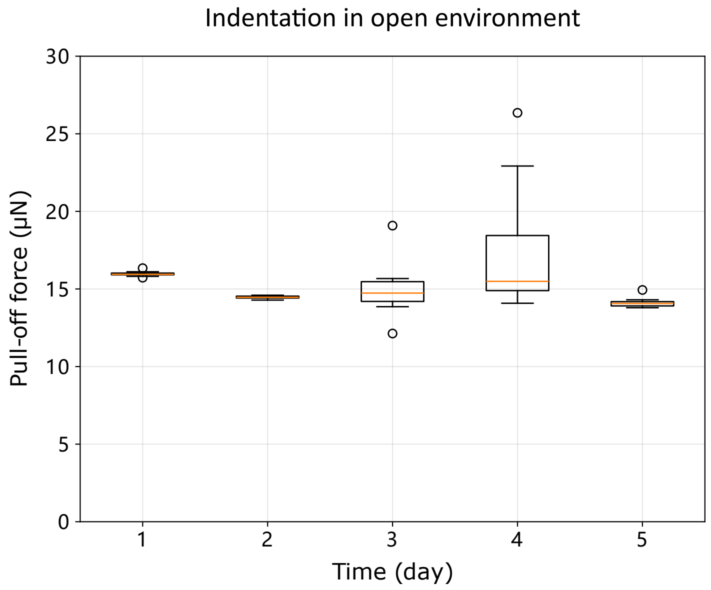

Fig. S7. Box graph of the pull-off force evolution in open environment

Supplementary Note 7: Geometry scaling for pick up

To evaluate the adaptability of the soft tip to different geometries and determine its potential limits for manipulable object dimensions, we conducted a series of approach–retract experiments on soft tips fabricated from IP-PDMS using the same parameters as our actuators. Two representative geometries were selected—cylindrical and spherical—to study how adhesion evolves on the soft tip contact surface across different scales.

As predicted by the DMT model (6) and the JKR model (8), the pull-off force between two surfaces (curved or flat) depends on the equivalent curvature radius, defined as $R_{eq} = \frac{R_1 R_2}{R_1 + R_2}$. Therefore, the curved-tip–flat-object configuration can be considered an equivalent substitute for the curved-object–flat-tip condition encountered in practical applications.

Based on this principle, we fabricated a series of hemispherical and cylindrical circular tips (tip diameter 15 µm, similar to that of the large SSA tip with R = 60 µm) with varying curvature diameters, as shown in Fig. S8a. The approach–retract experiments were carried out using FEMTO Tools (FT-S20000 NI, Si flat punch, 50 nN resolution). The Si flat tip approached the soft tip at a velocity of 0.2 µm/s. Upon reaching a preload of 1 µN, it was held in contact for 4 s before retracting at 0.1 µm/s until pull-off. Each experiment was repeated ten times for consistency.

According to the DMT model, the pull-force for the hemispherical contact should be predicted as:

$$F_{PO} = 2\pi\Delta\gamma_{Si/IP-PDMS}R_{eq} \quad [8]$$

This relation predicts a linear scaling of the pull-off force with respect to dimension. The model appears to hold well for small dimensions (≤50 µm), but as the size increases, a saturation effect begins to emerge, as shown in Fig.S8**b**. Such effect could be easily explained if we take into account the assumption governing the DMT model: the adhesive force outside the contact surface would strech the elastic hemisphere until an equilibrium is reached. But if the deformed contact area reaches its limit (the area of the tip) before an equilibrium is reached, then the adhesion could not reach its full potential during pull-off. Within this limit, as the curvature diameter increases, the contact between two tips would converge to a plane-plane contact, thus explaining the saturation pull-off force. A similar trend is observed for cylindrical contacts, as shown in Fig.S8**c**. In this case, the DMT model predicts that the scaling of the pull-off force with curvature radius for a fixed cylinder length follows a $\propto \sqrt{R_{eq}}$ dependence rather than a linear one. However, since the contact area projects onto a circular region, the model becomes more complex. For simplicity, we use the linear approximation observed in Fig.S8**d** to describe the scaling phenomenon. The similar saturation values obtained for both geometries further corroborate this interpretation.

From these observations, we conclude that for our soft tip applied on the SSA (R = 60 µm), geometric variation does not significantly impact the adhesion force until the curvature diameter falls below a threshold of approximately 100 µm. Beyond this threshold, the adhesion force decreases approximately linearly. Based on our analysis, this threshold would decrease as the tip area decreases, providing sufficient flexibility in manipulatable object dimensions to support our claim of universal pick-and-place capability.

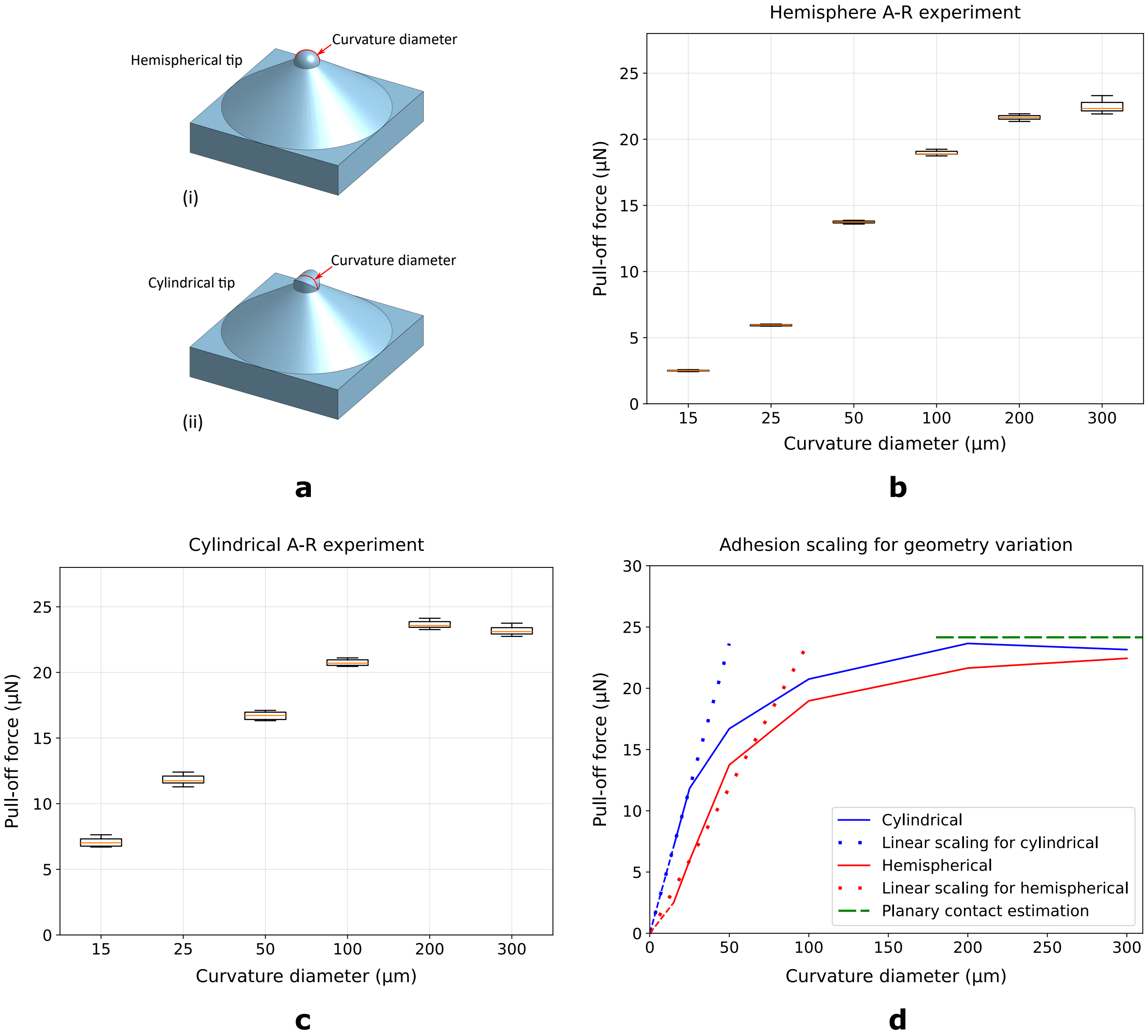

Fig. S8. Adhesion characterization of different contact geometry. a (i) Schematics of the IP-PDMS pad with hemispherical tip. (ii) Schematics of the IP-PDMS pad with cylindrical tip. **b** Box graph of the pull-off force evolution on hemispherical contact during 10 repetitions. **c** Box graph of the pull-off force evolution on cylindrical contact during 10 repetitions. **d** Pull-off force evolution of the two contact geometries along scale.

Movie S1. Two working modes (snap mode and continuous mode) achieved on two actuator sizes (R=60 µm and 15 µm, respectively).

Movie S2. Pick-and-release of a 200×200×58 µm Si chip and pick-and-place of a 200×200×25 µm IP-S polymer chip. Both achieved with a R=60 µm actuator on a glass substrate.

Movie S3. Universal pick-and-place. The pick-and-places of a 100 µm diameter glass bead and a 500 µm long, 25 µm diameter gold wire. Both achieved with a R=15 µm actuator on a glass substrate.

Movie S4. Mid-air manipulation of a 100×100×100 µm Ip-S cube deposited with Cr. The cube is first picked with the protruded IP-PDMS tip. Another actuator made another contact on the side after the first actuator retracted into orientation mode. After establishing resilient contacts on both sides, the first actuator fully retracts to release the cube. After a 90° turn, the cube is placed onto a Cr-deposited non-adhesive substrate.

Movie S5. Assembly and detachment of the release mask. The release mask is attached to the microgripper (R=15 µm) with adhesion, and then released onto an adhesive substrate (double-sided duct tape).

References

1. B Gorissen, D Melancon, N Vasios, M Torbati, K Bertoldi, Inflatable soft jumper inspired by shell snapping. *Sci. Robotics* **5**, eabb1967 (2020).
2. J Marthelot, F Lopez Jimenez, A Lee, J Hutchinson, P Reis, Buckling of a pressurized hemispherical shell subjected to a probing force. *J. Appl. Mech.* **84** (2017).
3. R Zoelly, Doctoral thesis (ETH Zurich, Zürich) (1915) Diss. Techn.Wiss. ETH Zürich, Nr. 146, 0000. Ref.: Meissner, E. ; Korref.: Kollros, L..
4. L Baumgarten, J Kierfeld, Shallow shell theory of the buckling energy barrier: From the pogorelov state to softening and imperfection sensitivity close to the buckling pressure. *Phys. Rev. E* **99** (2019).
5. PFJ van Altena, A Accardo, Micro 3d printing elastomeric ip-pdms using two-photon polymerisation: A comparative analysis of mechanical and feature resolution properties. *Polymers* **15** (2023).
6. B Derjaguin, V Muller, Y Toporov, Effect of contact deformations on the adhesion of particles. *J. Colloid Interface Sci.* **53**, 314–326 (1975).
7. CJ van Oss, Chapter two - the apolar and polar properties of liquid water and other condensed-phase materials in *The Properties of Water and their Role in Colloidal and Biological Systems*, Interface Science and Technology, ed. CJ van Oss. (Elsevier) Vol. 16, pp. 13–30 (2008).
8. K Johnson, K Kendall, A Roberts, Surface energy and contact of elastic solids. *Proc. The Royal Soc. A: Math. Phys. Eng. Sci.* **324**, 301–313 (1971).
